# Supplementary material for: Sugar-sweetened beverage consumption from 1998–2017: Findings from the health behaviour in school-aged children/school health research network in Wales
Source: PLoS One. 2021 Apr 14;16(4):e0248847. doi: 10.1371/journal.pone.0248847 (PMC8046241; doi:10.1371/journal.pone.0248847)
Supplement: S1 File — (DOCX) [file pone.0248847.s001.docx]

**S1 File**– Information on missing data

Full Information Maximum Likelihood (FIML) was an optimum method to handle the missing data, the statistical capacity of Stata 15 was unable to use FIML in non-linear models. In addition, the majority of the missingness attributed to the variables were missingness by design rather than non-response (i.e. no questions on socioeconomic status until 2002). The variables of year, gender and school year did not exceed 5% missingness, and hence bias due to missing data is very unlikely from these variables. However, in some years, socioeconomic status did exceed the rule of thumb (14% missing in 2009 and 12% in 2015).
