# Supplementary material for: Sugar-sweetened beverage consumption from 1998–2017: Findings from the health behaviour in school-aged children/school health research network in Wales
Source: PLoS One. 2021 Apr 14;16(4):e0248847. doi: 10.1371/journal.pone.0248847 (PMC8046241; doi:10.1371/journal.pone.0248847)
Supplement: S1 Table — (DOCX) [file pone.0248847.s002.docx]

|  | **1998** | **2000** | **2002** | **2004** | **2006** | **2009** | **2013** | **2015** | **2017** | **Total** | **Missing** |
| --- | --- | --- | --- | --- | --- | --- | --- | --- | --- | --- | --- |
| **Gender** |  |  |  |  |  |  |  |  |  |  | 2337 (1%) |
| **Boy** | 2,063 | 1,800 | 2,116 | 3,425 | 2,172 | 4,594 | 3,743 | 15,554 | 50,452 | 85,919 |  |
|  | *50.4%* | *51.5%* | *51.7%* | *48.5%* | *49.3%* | *50.2%* | *50.9%* | *48.0%* | *49.5%* | *49.39%* |  |
| **Girl** | 2,034 | 1,695 | 1,974 | 3,631 | 2,237 | 4,565 | 3,607 | 16,837 | 51,458 | 88,038 |  |
|  | *49.7%* | *48.5%* | *48.3%* | *51.5%* | *50.7%* | *49.8%* | *49.1%* | *52.0%* | *50.5%* | *50.6%* |  |
| **School Year** | |  |  |  |  |  |  |  |  |  | 0 (0%) |
| **Year 7** | 1,381 | 1,174 | 1,463 | 1,502 | 1,527 | 1,923 | 1,546 | 7,208 | 22,634 | 40,358 |  |
|  | *33.7%* | *33.6%* | *35.7%* | *21.3%* | *34.6%* | *20.9%* | *21.0%* | *22.3%* | *21.8%* | *22.9%* |  |
| **Year 8** | 0 | 0 | 0 | 1,465 | 0 | 2,026 | 1,547 | 7,008 | 22,421 | 34,467 |  |
|  | *0.0%* | *0.0%* | *0.0%* | *20.8%* | *0.0%* | *22.0%* | *21.0%* | *21.6%* | *21.6%* | *19.6%* |  |
| **Year 9** | 1,433 | 1,237 | 1,433 | 1,433 | 1,534 | 1,908 | 1,565 | 6,449 | 22,208 | 39,200 |  |
|  | *35.0%* | *35.4%* | *34.9%* | *20.3%* | *34.8%* | *20.8%* | *21.2%* | *19.9%* | *21.4%* | *22.3%* |  |
| **Year 10** | 0 | 0 | 0 | 1,408 | 0 | 1,687 | 1,413 | 6,358 | 19,704 | 30,570 |  |
|  | *0.0%* | *0.0%* | *0.0%* | *20.0%* | *0.0%* | *18.4%* | *19.2%* | *19.6%* | *19.0%* | *17.4%* |  |
| **Year 11** | 1,283 | 1,084 | 1,208 | 1,249 | 1,348 | 1,650 | 1,305 | 5,368 | 17,004 | 31,499 |  |
|  | *31.3%* | *31.0%* | *29.4%* | *17.7%* | *30.6%* | *18.0%* | *17.7%* | *16.6%* | *16.4%* | *17.9%* |  |
| **Socioeconomic Status** | |  |  |  |  |  |  |  |  |  | 14,315 (8%) |
| **High** | 0 | 0 | 1,703 | 3,268 | 2,137 | 3,308 | 3,163 | 13,531 | 51,770 | 78,880 |  |
|  | *0.0%* | *0.0%* | *46.7%* | *51.2%* | *51.7%* | *42.0%* | *44.2%* | *47.3%* | *49.8%* | *48.8%* |  |
| **Low** | 0 | 0 | 1,945 | 3,113 | 1,996 | 4,574 | 3,989 | 15,081 | 52,201 | 82,999 |  |
|  | *0.0%* | *0.0%* | *53.3%* | *48.8%* | *48.3%* | *58.0%* | *55.8%* | *52.7%* | *50.2%* | *51.2%* |  |

**S1 Table.** Sample characteristics of the study participants (11–16 years) between 1998 and 2017 (n=176,094)
