# Supplementary material for: Sugar-sweetened beverage consumption from 1998–2017: Findings from the health behaviour in school-aged children/school health research network in Wales
Source: PLoS One. 2021 Apr 14;16(4):e0248847. doi: 10.1371/journal.pone.0248847 (PMC8046241; doi:10.1371/journal.pone.0248847)
Supplement: S2 Table — (DOCX) [file pone.0248847.s003.docx]

| **Boys SSB consumption over-time** | | | | | | | | | | |
| --- | --- | --- | --- | --- | --- | --- | --- | --- | --- | --- |
|  | **1998** | **2000** | **2002** | **2004** | **2006** | **2009** | **2013** | **2015** | **2017** | **Total** |
| **Never or less than weekly use** | 112 | 117 | 223 | 440 | 404 | 898 | 819 | 3,632 | 11,939 | 18,584 |
|  | *5%* | *7%* | *11%* | *13%* | *19%* | *20%* | *22%* | *24%* | *24%* | *22%* |
| **Weekly use** | 693 | 547 | 1,063 | 1,757 | 1,091 | 2,476 | 2,142 | 8,462 | 27,417 | 45,648 |
|  | *34%* | *31%* | *51%* | *52%* | *50%* | *54%* | *57%* | *56%* | *55%* | *54%* |
| **Daily use** | 1,238 | 1,111 | 790 | 1,186 | 669 | 1,209 | 767 | 3,052 | 10,147 | 20,169 |
|  | *61%* | *63%* | *38%* | *35%* | *31%* | *26%* | *21%* | *20%* | *21%* | *24%* |
| **Total** | 2,043 | 1,775 | 2,076 | 3,383 | 2,164 | 4,583 | 3,728 | 15,146 | 49,503 | 84,401 |

| **Girls SSB consumption over-time** | | | | | | | | | | |
| --- | --- | --- | --- | --- | --- | --- | --- | --- | --- | --- |
|  | **1998** | **2000** | **2002** | **2004** | **2006** | **2009** | **2013** | **2015** | **2017** | **Total** |
| **Never or less than weekly use** | 185 | 222 | 275 | 726 | 598 | 1,268 | 1,018 | 5,481 | 17,355 | 27,128 |
|  | *9%* | *13%* | *14%* | *20%* | *27%* | 28% | 28% | *33%* | *34%* | *31%* |
| **Weekly use** | 736 | 584 | 978 | 1812 | 1046 | 2,320 | 1,886 | 8,276 | 25,115 | 42,753 |
|  | *37%* | *35%* | *50%* | *50%* | *47%* | *51%* | *52%* | *50%* | *49%* | *49%* |
| **Daily use** | 1092 | 876 | 704 | 1057 | 590 | 975 | 694 | 2,801 | 8,291 | 17,080 |
|  | *54%* | 52% | 36% | 29% | 26% | 21% | 19% | 17% | 16% | 20% |
| **Total** | 2,013 | 1,682 | 1,957 | 3,595 | 2,234 | 4,563 | 3,598 | 16,558 | 50,761 | 86,961 |

**S2 Table.** Boys and Girls SSB consumption over-time
