# Supplementary material for: Sugar-sweetened beverage consumption from 1998–2017: Findings from the health behaviour in school-aged children/school health research network in Wales
Source: PLoS One. 2021 Apr 14;16(4):e0248847. doi: 10.1371/journal.pone.0248847 (PMC8046241; doi:10.1371/journal.pone.0248847)
Supplement: S3 Table — (DOCX) [file pone.0248847.s004.docx]

| **Boys ED consumption over-time** | | | | |  | **Girls ED consumption over-time** | | | | |
| --- | --- | --- | --- | --- | --- | --- | --- | --- | --- | --- |
|  | **2013** | **2015** | **2017** | **Total** |  |  | **2013** | **2015** | **2017** | **Total** |
| **Never or less than weekly use** | 2477 | 10443 | 36432 | 49352 |  | **Never or less than weekly use** | 2828 | 13276 | 42594 | 58698 |
|  | *66%* | *69%* | *74%* | *72%* |  |  | *79%* | *80%* | *84%* | *83%* |
| **Weekly use** | 1013 | 3664 | 9627 | 14304 |  | **Weekly use** | 618 | 2543 | 6111 | 9272 |
|  | *27%* | *24%* | *19%* | *21%* |  |  | *17%* | *15%* | *12%* | *13%* |
| **Daily use** | 240 | 1039 | 3397 | 4676 |  | **Daily use** | 152 | 728 | 2133 | 3013 |
|  | *6%* | *7%* | *7%* | *7%* |  |  | *4%* | *4%* | *4%* | *4%* |
| **Total** | 3730 | 15146 | 49,456 | 68332 |  | Total | 3598 | 16547 | 50,838 | 70983 |

**S3 Table.** Boys and Girls ED consumption over-time
