# Supplementary material for: Sugar-sweetened beverage consumption from 1998–2017: Findings from the health behaviour in school-aged children/school health research network in Wales
Source: PLoS One. 2021 Apr 14;16(4):e0248847. doi: 10.1371/journal.pone.0248847 (PMC8046241; doi:10.1371/journal.pone.0248847)
Supplement: S4 Table — (DOCX) [file pone.0248847.s005.docx]

| **High SES SSB consumption over-time** | | | | | | | | |
| --- | --- | --- | --- | --- | --- | --- | --- | --- |
|  | **2002** | **2004** | **2006** | **2009** | **2013** | **2015** | **2017** | **Total** |
| **Never or less than weekly use** | 212 | 565 | 498 | 876 | 851 | 4,136 | 15,910 | 23,048 |
|  | *13%* | *17%* | *23%* | *27%* | *27%* | *31%* | *31%* | *30%* |
| **Weekly use** | 854 | 1,680 | 1,056 | 1,701 | 1,790 | 7,199 | 26,866 | 41,146 |
|  | *51%* | *52%* | *49%* | *51%* | *57%* | *54%* | *53%* | *53%* |
| **Daily use** | 618 | 996 | 580 | 728 | 521 | 2,073 | 8,257 | 13,773 |
|  | *37%* | *31%* | *27%* | *22%* | *16%* | *15%* | *16%* | *18%* |
| **Total** | 1,684 | 3,241 | 2,134 | 3,305 | 3,162 | 13,408 | 51,033 | 77,967 |

| **Low SES SSB consumption over-time** | | | | | | | | |
| --- | --- | --- | --- | --- | --- | --- | --- | --- |
|  | **2002** | **2004** | **2006** | **2009** | **2013** | **2015** | **2017** | **Total** |
| **Never or less than weekly use** | 239 | 496 | 428 | 1,004 | 935 | 4,031 | 13,913 | 21,046 |
|  | *12%* | *16%* | *22%* | *22%* | *24%* | *27%* | *27%* | *26%* |
| **Weekly use** | 967 | 1,555 | 949 | 2,440 | 2,136 | 7,815 | 26,455 | 42,317 |
|  | *51%* | *51%* | *48%* | *53%* | *54%* | *52%* | *52%* | *52%* |
| **Daily use** | 709 | 1020 | 613 | 1124 | 903 | 3,063 | 10,681 | 18,113 |
|  | *37%* | *33%* | *31%* | *25%* | *23%* | *21%* | *21%* | *22%* |
| **Total** | 1,915 | 3,071 | 1,990 | 4,568 | 3,974 | 14,909 | 51,049 | 81,476 |

**S4 Table.** High and Low SES SSB consumption over-time
