# Supplementary material for: Sugar-sweetened beverage consumption from 1998–2017: Findings from the health behaviour in school-aged children/school health research network in Wales
Source: PLoS One. 2021 Apr 14;16(4):e0248847. doi: 10.1371/journal.pone.0248847 (PMC8046241; doi:10.1371/journal.pone.0248847)
Supplement: S5 Table — (DOCX) [file pone.0248847.s006.docx]

| **High SES ED consumption over-time** | | | | |  | **Low SES ED consumption over-time** | | | | |
| --- | --- | --- | --- | --- | --- | --- | --- | --- | --- | --- |
|  | **2013** | **2015** | **2017** | **Total** |  |  | **2013** | **2015** | **2017** | **Total** |
| **Never or less than weekly use** | 2373 | 10417 | 41110 | 53900 |  | **Never or less than weekly use** | 2792 | 10929 | 39061 | 52782 |
|  | *75%* | *78%* | *81%* | *80%* |  |  | *70%* | *73%* | *77%* | *75%* |
| **Weekly use** | 646 | 2405 | 7480 | 10531 |  | **Weekly use** | 942 | 3099 | 8609 | 12650 |
|  | *20%* | *18%* | *15%* | *16%* |  |  | *24%* | *21%* | *17%* | *18%* |
| **Daily use** | 143 | 580 | 2456 | 3179 |  | **Daily use** | 242 | 871 | 3392 | 4505 |
|  | *5%* | *4%* | *5%* | *5%* |  |  | *6%* | *6%* | *7%* | *6%* |
| **Total** | 3162 | 13402 | 51,046 | 67610 |  | **Total** | 3976 | 14899 | 51,062 | 69937 |

**S5 Table.** High and Low SES ED consumption over-time
