# Supplementary material for: Sugar-sweetened beverage consumption from 1998–2017: Findings from the health behaviour in school-aged children/school health research network in Wales
Source: PLoS One. 2021 Apr 14;16(4):e0248847. doi: 10.1371/journal.pone.0248847 (PMC8046241; doi:10.1371/journal.pone.0248847)
Supplement: S6 Table — (DOCX) [file pone.0248847.s007.docx]

| **Year 7's SSB consumption over-time** | | | | | | | | | | |
| --- | --- | --- | --- | --- | --- | --- | --- | --- | --- | --- |
|  | **1998** | **2000** | **2002** | **2004** | **2006** | **2009** | **2013** | **2015** | **2017** | **Total** |
| **Never or less than weekly use** | 86 | 114 | 178 | 264 | 393 | 513 | 387 | 2,296 | 7,000 | 11,231 |
|  | *6%* | *10%* | *12%* | *18%* | *26%* | *27%* | *25%* | *33%* | *32%* | *28%* |
| **Weekly use** | 458 | 373 | 770 | 792 | 765 | 1,022 | 884 | 3,690 | 11,459 | 20,213 |
|  | *34%* | *32%* | *54%* | *54%* | *50%* | *53%* | *58%* | *52%* | *52%* | *51%* |
| **Daily use** | 823 | 672 | 488 | 416 | 364 | 383 | 265 | 1,079 | 3,636 | 8,126 |
|  | *60%* | *58%* | *34%* | *28%* | *24%* | *20%* | *17%* | *15%* | *16%* | *21%* |
| **Total** | 1,367 | 1,159 | 1,436 | 1,472 | 1,522 | 1,918 | 1,536 | 7,065 | 22,095 | 39,570 |

| **Year 7's ED consumption over-time** | | | | |
| --- | --- | --- | --- | --- |
|  | **2013** | **2015** | **2017** | **Total** |
| **Never or less than weekly use** | 1,167 | 5,685 | 18,439 | 25,291 |
|  | *76%* | *81%* | *83%* | *82%* |
| **Weekly use** | 305 | 1,093 | 2,668 | 4,066 |
|  | *20%* | *15%* | *12%* | *13%* |
| **Daily use** | 65 | 284 | 1,006 | 1,355 |
|  | *4%* | *4%* | *5%* | *4%* |
| **Total** | 1,537 | 7,062 | 22,113 | 30,712 |

**S6 Table.** Year 7’s SSB and ED consumption over-time
