# Supplementary material for: Sugar-sweetened beverage consumption from 1998–2017: Findings from the health behaviour in school-aged children/school health research network in Wales
Source: PLoS One. 2021 Apr 14;16(4):e0248847. doi: 10.1371/journal.pone.0248847 (PMC8046241; doi:10.1371/journal.pone.0248847)
Supplement: S7 Table — (DOCX) [file pone.0248847.s008.docx]

| **Year 8's SSB consumption over-time** | | | | | | |
| --- | --- | --- | --- | --- | --- | --- |
|  | **2004** | **2009** | **2013** | **2015** | **2017** | **Total** |
| **Never or less than weekly use** | 237 | 502 | 376 | 2,095 | 6,382 | 9,592 |
|  | *16%* | *25%* | *24%* | *31%* | *29%* | *28%* |
| **Weekly use** | 781 | 1083 | 860 | 3,603 | 11,784 | 18,111 |
|  | *54%* | *54%* | *56%* | *53%* | *54%* | *53%* |
| **Daily use** | 434 | 434 | 307 | 1,155 | 3,829 | 6,159 |
|  | *30%* | *22%* | *20%* | *17%* | *17%* | *18%* |
| **Total** | 1,452 | 2,019 | 1,543 | 6,853 | 21,995 | 33,862 |

| **Year 8's ED consumption over-time** | | | | |
| --- | --- | --- | --- | --- |
|  | **2013** | **2015** | **2017** | **Total** |
| **Never or less than weekly use** | 1,133 | 5,209 | 17,562 | 23,904 |
|  | *73%* | *76%* | *80%* | *79%* |
| **Weekly use** | 320 | 1,291 | 3,305 | 4,916 |
|  | *21%* | *19%* | *15%* | *16%* |
| **Daily use** | 90 | 345 | 1,115 | 1,550 |
|  | *6%* | *5%* | *5%* | *5%* |
| **Total** | 1,543 | 6,845 | 21,982 | 30,370 |

**S7 Table.** Year 8’s SSB and ED consumption over-time
