# Supplementary material for: Sugar-sweetened beverage consumption from 1998–2017: Findings from the health behaviour in school-aged children/school health research network in Wales
Source: PLoS One. 2021 Apr 14;16(4):e0248847. doi: 10.1371/journal.pone.0248847 (PMC8046241; doi:10.1371/journal.pone.0248847)
Supplement: S8 Table — (DOCX) [file pone.0248847.s009.docx]

| **Year 9's SSB consumption over-time** | | | | | | | | | | |
| --- | --- | --- | --- | --- | --- | --- | --- | --- | --- | --- |
|  | **1998** | **2000** | **2002** | **2004** | **2006** | **2009** | **2013** | **2015** | **2017** | **Total** |
| **Never or less than weekly use** | 88 | 110 | 177 | 226 | 316 | 428 | 358 | 1,677 | 6,119 | 9,499 |
|  | *6%* | *9%* | *13%* | *16%* | *21%* | *22%* | *23%* | *27%* | *28%* | 25% |
| **Weekly use** | 505 | 391 | 681 | 719 | 720 | 980 | 854 | 3,409 | 11,429 | 19,688 |
|  | *35%* | *32%* | *48%* | *51%* | *47%* | *51%* | *55%* | *54%* | *52%* | *51%* |
| **Daily use** | 832 | 724 | 553 | 471 | 493 | 500 | 348 | 1,220 | 4,259 | 9,400 |
|  | *58%* | *59%* | *39%* | *33%* | *32%* | *26%* | *22%* | *19%* | *20%* | *24%* |
| **Total** | 1,425 | 1,225 | 1,411 | 1,416 | 1,529 | 1,908 | 1,560 | 6,306 | 21,807 | 38,587 |

| **Year 9's ED consumption over-time** | | | | |
| --- | --- | --- | --- | --- |
|  | **2013** | **2015** | **2017** | **Total** |
| **Never or less than weekly use** | 1,105 | 4,505 | 16,647 | 22,257 |
|  | *71%* | *71%* | *76%* | *75%* |
| **Weekly use** | 360 | 1,409 | 3,792 | 5,561 |
|  | *23%* | *22%* | *17%* | *19%* |
| **Daily use** | 95 | 403 | 1,381 | 1,879 |
|  | *6%* | *6%* | *6%* | *6%* |
| **Total** | 1,560 | 6,317 | 21,820 | 29,697 |

**S8 Table.** Year 9’s SSB and ED consumption over-time
