# Supplementary material for: Sugar-sweetened beverage consumption from 1998–2017: Findings from the health behaviour in school-aged children/school health research network in Wales
Source: PLoS One. 2021 Apr 14;16(4):e0248847. doi: 10.1371/journal.pone.0248847 (PMC8046241; doi:10.1371/journal.pone.0248847)
Supplement: S9 Table — (DOCX) [file pone.0248847.s010.docx]

| **Year 10's SSB consumption over-time** | | | | | | |
| --- | --- | --- | --- | --- | --- | --- |
|  | **2004** | **2009** | **2013** | **2015** | **2017** | **Total** |
| **Never or less than weekly use** | 238 | 371 | 400 | 1,578 | 5,526 | 8,113 |
|  | *17%* | *22%* | *28%* | *25%* | *28%* | *27%* |
| **Weekly use** | 682 | 863 | 745 | 3,329 | 10,031 | 15,650 |
|  | *49%* | *51%* | *53%* | *53%* | *52%* | *52%* |
| **Daily use** | 478 | 452 | 265 | 1,336 | 3,856 | 6,387 |
|  | *34%* | *27%* | *19%* | *21%* | *20%* | *21%* |
| **Total** | 1,398 | 1,686 | 1,410 | 6,243 | 19,413 | 30,150 |

| **Year 10's ED consumption over-time** | | | | |
| --- | --- | --- | --- | --- |
|  | **2013** | **2015** | **2017** | **Total** |
| **Never or less than weekly use** | 1,001 | 4,443 | 14,651 | 20,095 |
|  | *71%* | *71%* | *75%* | *74%* |
| **Weekly use** | 341 | 1,377 | 3,442 | 5,160 |
|  | *24%* | *22%* | *18%* | *19%* |
| **Daily use** | 68 | 418 | 1,327 | 1,813 |
|  | *5%* | *7%* | *7%* | *7%* |
| **Total** | 1,410 | 6,238 | 19,420 | 27,068 |

**S9 Table.** Year 10’s SSB and ED consumption over-time
