# Supplementary material for: Sugar-sweetened beverage consumption from 1998–2017: Findings from the health behaviour in school-aged children/school health research network in Wales
Source: PLoS One. 2021 Apr 14;16(4):e0248847. doi: 10.1371/journal.pone.0248847 (PMC8046241; doi:10.1371/journal.pone.0248847)
Supplement: S10 Table — (DOCX) [file pone.0248847.s011.docx]

| **Year 11's SSB consumption over-time** | | | | | | | | | | |
| --- | --- | --- | --- | --- | --- | --- | --- | --- | --- | --- |
|  | **1998** | **2000** | **2002** | **2004** | **2006** | **2009** | **2013** | **2015** | **2017** | **Total** |
| **Never or less than weekly use** | 123 | 115 | 145 | 201 | 293 | 362 | 320 | 1,467 | 4,796 | 7,822 |
|  | *10%* | *11%* | *12%* | *16%* | *22%* | *22%* | *25%* | *28%* | *29%* | *25%* |
| **Weekly use** | 466 | 367 | 597 | 596 | 652 | 863 | 700 | 2,707 | 8,618 | 15,566 |
|  | *37%* | *34%* | *50%* | *48%* | *48%* | *52%* | *54%* | *52%* | *51%* | *50%* |
| **Daily use** | 675 | 591 | 458 | 444 | 402 | 424 | 282 | 1,063 | 3,358 | 7,697 |
|  | *53%* | *55%* | *38%* | *36%* | *30%* | *26%* | *22%* | *20%* | *20%* | *25%* |
| **Total** | 1,264 | 1,073 | 1,200 | 1,241 | 1,347 | 1,649 | 1,302 | 5,237 | 16,772 | 31,085 |

| **Year 11's ED consumption over-time** | | | | |
| --- | --- | --- | --- | --- |
|  | **2013** | **2015** | **2017** | **Total** |
| **Never or less than weekly use** | 912 | 3,877 | 12,872 | 17,661 |
|  | *70%* | *74%* | *77%* | *76%* |
| **Weekly use** | 315 | 1,037 | 2,882 | 4,234 |
|  | *24%* | *20%* | *17%* | *18%* |
| **Daily use** | 76 | 317 | 1,019 | 1,412 |
|  | *6%* | *6%* | *6%* | *6%* |
| **Total** | 1,303 | 5,231 | 16,773 | 23,307 |

**S10 Table.** Year 11’s SSB and ED consumption over-time
