# Supplementary material for: Sugar-sweetened beverage consumption from 1998–2017: Findings from the health behaviour in school-aged children/school health research network in Wales
Source: PLoS One. 2021 Apr 14;16(4):e0248847. doi: 10.1371/journal.pone.0248847 (PMC8046241; doi:10.1371/journal.pone.0248847)
Supplement: S11 Table — (DOCX) [file pone.0248847.s012.docx]

|  | **Sugary drink** | | | |
| --- | --- | --- | --- | --- |
| **Energy drink** | **Never or less than weekly** | **Weekly use** | **Daily use** | **Total** |
|  |  |  |  |  |
| **Never or less than weekly** | 38,157 | 56,357 | 14,154 | 108,668 |
|  | *94%* | *76%* | *54%* | *77%* |
| **Weekly use** | 2,022 | 15,784 | 6,045 | 23,851 |
|  | *5%* | *21%* | *23%* | *17%* |
| **Daily use** | 497 | 1,649 | 5,805 | 7,951 |
|  | *1%* | *2%* | *22%* | *6%* |
| **Total** | 40,676 | 73,790 | 26,004 | 140,470 |

**S11 Table.** Cross-tabulation of sugary drink consumption and energy drink consumption
