# Supplementary material for: Sugar-sweetened beverage consumption from 1998–2017: Findings from the health behaviour in school-aged children/school health research network in Wales
Source: PLoS One. 2021 Apr 14;16(4):e0248847. doi: 10.1371/journal.pone.0248847 (PMC8046241; doi:10.1371/journal.pone.0248847)
Supplement: S12 Table — (DOCX) [file pone.0248847.s013.docx]

| **Interaction variable** | **Sugary drink** | | **Energy drink** | |
| --- | --- | --- | --- | --- |
|  | Never | Weekly | Never | Weekly |
| **Gender*Year** | 1.01 (0.98 - 1.05) | 1.00 (0.97 - 1.03) | 1.09 (0.88 - 1.35) | 1.12 (0.89 - 1.41) |
| **School Year*Year** | 1.00 (0.98 – 1.01) | 1.01 (0.99 - 1.02) | 0.98 (0.89 - 1.09) | 1.05 (0.93 - 1.17) |
| **Socioeconomic Status*Year** | **0.91 (0.88 - 0.95)** | **0.92 (0.89 - 0.95)** | 0.95 (0.77 - 1.17) | 0.92 (0.73 - 1.16) |

**S12 Table.** Interactions adjusted for other confounders; gender, school year and socioeconomic status (estimates in bold = p<0.05)
