# Supplementary material for: Sugar-sweetened beverage consumption from 1998–2017: Findings from the health behaviour in school-aged children/school health research network in Wales
Source: PLoS One. 2021 Apr 14;16(4):e0248847. doi: 10.1371/journal.pone.0248847 (PMC8046241; doi:10.1371/journal.pone.0248847)
Supplement: S13 Table — (DOCX) [file pone.0248847.s014.docx]

|  |  |  |  |  |  |  |  |  |  |  |
| --- | --- | --- | --- | --- | --- | --- | --- | --- | --- | --- |
| **Categories (<= 2000)** | **1998** | **2000** | **Categories (> 2000)** | **2002** | **2004** | **2006** | **2009** | **2013** | **2015** | **2017** |
| **Never or less than weekly** | 297 | 339 | **Never** | 143 | 360 | 348 | 721 | 627 | 2640 | 11208 |
|  | *7%* | *10%* |  | *4%* | *5%* | *8%* | *8%* | *9%* | *8%* | *10%* |
| **Weekly/Once a week** | 216 | 190 | **Less than once a week** | 357 | 806 | 654 | 1455 | 1214 | 7298 | 21377 |
|  | *5%* | *6%* |  | *9%* | *11%* | *15%* | *16%* | *17%* | *21%* | *19%* |
| **2 - 4 a week** | 589 | 478 | **Once a week** | 382 | 733 | 564 | 1413 | 1187 | 6604 | 20250 |
|  | *15%* | *14%* |  | *9%* | *10%* | *13%* | *15%* | *16%* | *19%* | *18%* |
| **5 - 6 a week** | 624 | 463 | **2 - 4 days a week** | 942 | 1624 | 966 | 2190 | 1868 | 8208 | 24498 |
|  | *15%* | *13%* |  | *23%* | *23%* | *22%* | *24%* | *25%* | *23%* | *22%* |
| **Daily** | 762 | 665 | **5 - 6 days a week** | 724 | 1213 | 607 | 1208 | 988 | 3326 | 12660 |
|  | *19%* | *19%* |  | *18%* | *17%* | *14%* | *13%* | *13%* | *9%* | *11%* |
| **Daily+** | 1568 | 1322 | **Once a day, every day** | 540 | 801 | 492 | 910 | 613 | 2759 | 9346 |
|  | *39%* | *38%* |  | *13%* | *11%* | *11%* | *10%* | *8%* | *8%* | *8%* |
| **Total** | 4056 | 3457 | **Every day, more than once** | 959 | 1442 | 767 | 1283 | 854 | 3503 | 10735 |
|  |  |  |  | *24%* | *20%* | *17%* | *14%* | *12%* | *10%* | *10%* |
|  |  |  | **Total** | 4047 | 6979 | 4398 | 9180 | 7351 | 34338 | 110074 |

**S13 Table.** SSB over-time before recoding
