# Supplementary material for: Sugar-sweetened beverage consumption from 1998–2017: Findings from the health behaviour in school-aged children/school health research network in Wales
Source: PLoS One. 2021 Apr 14;16(4):e0248847. doi: 10.1371/journal.pone.0248847 (PMC8046241; doi:10.1371/journal.pone.0248847)
Supplement: S14 Table — (DOCX) [file pone.0248847.s015.docx]

|  |  |  |  |  |  |  |  |  |  |
| --- | --- | --- | --- | --- | --- | --- | --- | --- | --- |
| **Categories** | **1998** | **2000** | **2002** | **2004** | **2006** | **2009** | **2013** | **2015** | **2017** |
| **Never or less than weekly** | 297 | 339 | 500 | 1166 | 1002 | 2176 | 1841 | 9938 | 32585 |
|  | *7%* | *10%* | *12%* | *17%* | *23%* | *24%* | *25%* | *29%* | *30%* |
| **Weekly** | 1429 | 1131 | 2048 | 3570 | 2137 | 4811 | 4043 | 18138 | 57408 |
|  | *35%* | *33%* | *51%* | *51%* | *49%* | *52%* | *55%* | *53%* | *52%* |
| **Daily or more** | 2330 | 1987 | 1499 | 2243 | 1259 | 2193 | 1467 | 6262 | 20081 |
|  | *57%* | *57%* | *37%* | *32%* | *29%* | *24%* | *20%* | *18%* | *18%* |
| **Total** | 4056 | 3457 | 4047 | 6979 | 4398 | 9180 | 7351 | 34338 | 110074 |

**S14 Table.** SSB over-time after recoding
