# Supplementary material for: Sugar-sweetened beverage consumption from 1998–2017: Findings from the health behaviour in school-aged children/school health research network in Wales
Source: PLoS One. 2021 Apr 14;16(4):e0248847. doi: 10.1371/journal.pone.0248847 (PMC8046241; doi:10.1371/journal.pone.0248847)
Supplement: S15 Table — (DOCX) [file pone.0248847.s016.docx]

**S15 Table.** ED over-time before recoding

|  | **2013** | **2015** | **2017** |
| --- | --- | --- | --- |
| **Never** | 3,693 | 18088 | 67262 |
|  | *50%* | *52%* | *60%* |
| **Less than once a week** | 1,625 | 7813 | 19729 |
|  | *22%* | *22%* | *18%* |
| **Once a week** | 751 | 3205 | 8030 |
|  | *10%* | *9%* | *7%* |
| **2 - 4 days a week** | 608 | 2401 | 5980 |
|  | *8%* | *7%* | *5%* |
| **5 - 6 days a week** | 282 | 961 | 2966 |
|  | *4%* | *3%* | *3%* |
| **Once a day, every day** | 169 | 725 | 2344 |
|  | *2%* | *2%* | *2%* |
| **Every day, more than once** | 225 | 1140 | 3796 |
|  | *3%* | *3%* | *3%* |
| **Total** | 7,353 | 34,333 | 110107 |
