# Supplementary material for: Sugar-sweetened beverage consumption from 1998–2017: Findings from the health behaviour in school-aged children/school health research network in Wales
Source: PLoS One. 2021 Apr 14;16(4):e0248847. doi: 10.1371/journal.pone.0248847 (PMC8046241; doi:10.1371/journal.pone.0248847)
Supplement: S16 Table — (DOCX) [file pone.0248847.s017.docx]

**S16 Table.** ED over-time after recoding

|  | **2013** | **2015** | **2017** |
| --- | --- | --- | --- |
| **Never or less than weekly** | 5318 | 25901 | 86991 |
|  | *72%* | *75%* | *79%* |
| **Weekly use** | 1641 | 6567 | 16976 |
|  | *22%* | *19%* | *15%* |
| **Daily use** | 394 | 1865 | 6140 |
|  | *5%* | *5%* | *6%* |
| **Total** | 7353 | 34333 | 110107 |
