# Supplementary figures and images for: Sugar-sweetened beverage consumption from 1998–2017: Findings from the health behaviour in school-aged children/school health research network in Wales
Source: PLoS One. 2021 Apr 14;16(4):e0248847. doi: 10.1371/journal.pone.0248847 (PMC8046241; doi:10.1371/journal.pone.0248847)

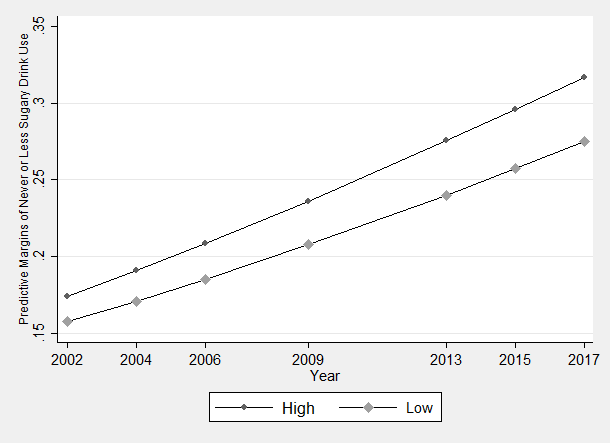

Supplement: S1 Fig — (TIF) [file pone.0248847.s018.tif]

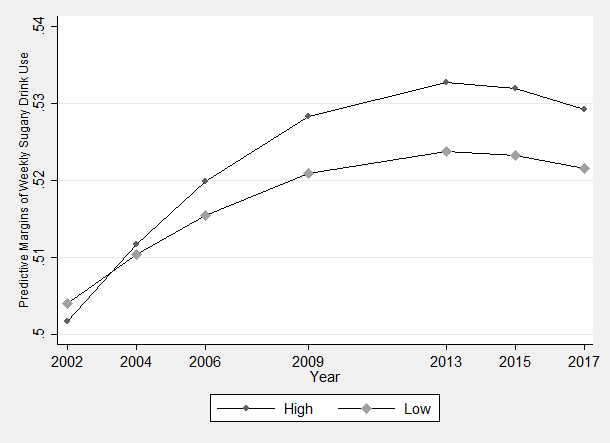

Supplement: S2 Fig — (TIF) [file pone.0248847.s019.tif]
