# Supplementary material for: Sugar-sweetened beverage consumption from 1998–2017: Findings from the health behaviour in school-aged children/school health research network in Wales
Source: PLoS One. 2021 Apr 14;16(4):e0248847. doi: 10.1371/journal.pone.0248847 (PMC8046241; doi:10.1371/journal.pone.0248847)
Supplement: Attachment — Submitted filename: Response to Reviewers.docx [file pone.0248847.s020.docx]

**PONE-D-20-21401
Sugar-sweetened beverage consumption from 1998-2017: findings from the health behaviour in school-aged children/school health research network in Wales**

Response to Reviewers

**Reviewer 1 comments**

1. Enjoyed reading this piece of work. Overall this is an interesting piece of work considering change of SSB and ED in adolescents using cross sectional data. There are a number of changes noted below for the authors to consider and a few questions to consider.

**Response**: Thank you for taking the time to review our paper. We have responded to your suggested changes and comments below.

Abstract:

2. Can you add some figures to back up key findings? Is there an overall conclusion for paper re implications for PH?

**Response**: Thank you for your suggestions. The following conclusion has been added “Given the established longer term impacts of SSB and ED consumption on adolescent health outcomes, urgent policy action is required to reduce overall consumption rates, with close attention to equity of impact throughout policy design and evaluation plans.” We have been mindful to limit our abstract word count in keeping with the journal requirements and are therefore unable to add figures throughout.

Intro:

3. Line 60: Rather than ‘it is linked to’ can you make this sentence a little clear to say consumption of these drinks are linked to dental erosion and Type II diabetes

**Response:** Thank you for your suggestion, the text has been amended to read ‘with consumption rates linked to an increased risk of type II diabetes [1] and dental erosion [2].

4. Line 62: Can you add an actual figure where you say ‘of which Eds make up a contribution’ as you have done for SSBs.

**Response**: Unfortunately, we are unable to locate an exact figure among the literature but we have added a follow-on sentence and accompanying reference to read “In 2017, one study found that 95% of EDs would receive a ‘red’ (high) label for sugars per serving [5].”

5. Line 68: Because you are considering both SSB and Eds can you also provide the context for SSBs re economic contribution.

**Response:** Thank you for your suggestion. The following sentence has been added: ‘It is estimated that 1 billion litres of soft drinks are produced globally each year [8] with the soft drinks industry contributing £11 billion to UK economic growth [9].’

6. Lines 100-102: The aims and objectives need to be made clearer and more specific for this paper. Make reference to the data you explored, the specific time periods, and also the key variables explored.

**Response**: Thank you for your suggestion. In line with reviewer 2’s comments also, we have updated the text to read “The aim of this study is to examine the consumption frequency of SSBs and EDs among 11-16 year olds over time. With use of national data collected between 1998 and 2017, we examined overall consumption and reports among sociodemographic subgroups. Data available for SSB consumption spanned a 20-year period (1998-2017) and ED consumption across five years (2013-2017).” (Lines 104-109)

7. Overall comment: As you start with SSB discussion – can you restructure the introduction to discuss aspects related to SSBs then EDs to make it more succinct and flow better. You tend to focus more on EDs than SSBs in parts of intro so the rationale in lines 98-99 only capture the ED aspect as opposed to the importance of exploring the effect of SSB consumption in this age group as well. Also, is this analysis based on total daily consumption of SSBs and EDs?

**Response**: Given that this is largely a matter of stylistic preference, and that the reviewer offers this as a suggestion rather than a necessary improvement, we have chosen not restructure the introduction. We have however, inserted additional information and restructured the study aims as requested above. We have also added the additional text “SSB and ED consumption among” (Line 99) to capture the importance of both consumption patterns.

Methods:

8. Lines 108-110: I appreciate you have referenced the data are appended as per previous studies and but can you provide more context? For example, how frequently are these surveys carried out between 1998-2017 – yearly, every 2 years etc?

**Response**: Thank you for your suggestion. We have added the following sentence: “Surveys were conducted approximately every two years from 1998 to 2017” (Lines 116-120) and “Over-time the SHRN survey sample size has grown due to the increasing number of schools in Wales agreeing to conduct the survey.” (Line 122).

9. Lines 107-110: I think the authors need to include more detail about the survey methods. In particular, the method used to obtain dietary data (FFQs). This can then be linked to the section on ‘limitations’ where they mention the reliance on self-reported data.

**Response**: Thank you for your suggestion. We have now updated the ‘study sample’ section as per your comments and those of Reviewer 2. As our study utilises secondary data, our SSB and ED consumption are based on existing survey questions (the details of these questions are currently outlined within our methods section; lines 140-158) with response categories streamlined in order to harmonise responses over-time.

10. Socio-demographics – this section includes more than that so may be better as participant characteristics as you have gender, year etc included

**Response**: Thank you for your suggestion. We feel the heading is reflective of the sociodemographic characteristics described (e.g. gender, age and family affluence) and have therefore opted not to make any changes to the sub-heading.

11. Line 116 and 121: FAS method, I’m not overly familiar with this method but can you explain why you only have two groups - low and high for SES effect? You should also note the limitations of this method for SES in the discussion.

**Response**: We have used the Family Affluence Scale split into groups for SES. This is a common technique and is used in many of the HBSC survey EU national reports (please see <https://www.euro.who.int/en/health-topics/Life-stages/child-and-adolescent-health/health-behaviour-in-school-aged-children-hbsc/publications/2020/spotlight-on-adolescent-health-and-well-being.-findings-from-the-20172018-health-behaviour-in-school-aged-children-hbsc-survey-in-europe-and-canada.-international-report.-volume-1.-key-findings>). The continuous score can be difficult to interpret, whereas a binary score can aid understanding and is easier to interpret statistically. However, the loss of detail could be a drawback from some researchers’ point of view and as a result we have acknowledged this as a limitation; “Third, the use of a binary measure of socioeconomic status may limit interpretations, despite being widely used.” (Line 424-425).

12. In this section you also mention SES was only available from 2002 – what did you use as SES categorisation for data pre-2002? Or was this excluded for these data? This needs noted.

**Response**: Thank you for your clarification. Yes, you are correct, we did not use any measure – they were excluded and no SES measure was used for 1998 and 2000. Therefore, within the RRR models these would be missing with estimates reported for 2002-2017 only. We have since reflected this point within our limitations “For example, socioeconomic data were not collected in 1998 and 2000 with SSB regression models estimated from 2002”. (Line 422)

13. Did the change in SES coding have any impact (2002 and then in 2013 changed – if not tested that’s okay but then need to note in limitations categorisation of SES did change between these time points)

**Response**: We thank the reviewer for this comment. Although previous papers do not specify that the change in measurement has made a difference, and our proportions do not differ over the years considerably, there is some evidence that the addition has led to the groups being more equally split. Therefore, we have added this to the limitations section: “For example, socioeconomic data were not collected in 1998 and 2000 with SSB regression models estimated from 2002 and data include two extra measures of socioeconomic status from 2013 onwards.” (Line 422-423).

14. Line 129: Here you mention in the first two survey years for SSBs - can you provide the actual survey years and periods you have from 1998-2017 (I see this in S1 table but it would help the reader to be included in the text) and be specific that the ED data only includes 2013-2017; that is quite different (4y verse 20y)

**Response**: Thank you for this suggestion, it is important that our paper is clear on this matter; we have added this in the study sample section as mentioned earlier; “Surveys were conducted approximately every two years from 1998 to 2017. Data are appended over the years to create a repeated cross-sectional dataset as in previous studies [27]; Data on SSBs were available from 1998-2017, and data on EDs from 2013-2017.” (Lines 116-118).

15. Line 164: change to ‘using’ Stata15

**Response**: Text has been amended.

Results:

16. Currently are lengthy. I’m not convinced all the supplementary tables are required as a lot are descriptive. A few suggestions would be to condense the descriptive stats and focus more on the overall key findings from the more complex model results, once you have factored in year, SES and gender. Otherwise they get lost.

**Response**: We thank the reviewer for this suggestion. However, as this is a survey which is used across Europe, we endeavour to make our research process very clear for other countries to consider in similar analyses to create cross-country comparisons. While there may be a few more supplementary tables than usual, we would like to retain them for other researchers’ purposes, particularly as the reader can choose to download the supplementary files if they so wish.

17. Also you have significant interactions – can you provide more detail/explanation on the interaction effects rather than just refer to a supplementary table – this is one of the supplementary tables that may be better in the results findings.

**Response**: We thank the reviewer for this comment. We decided to not include the interaction table as most interactions were not statistically significant and stable over-time, e.g., RRR of 1.00 or 1.01 for SBBs. Due to this, it was straightforward to communicate the statistically significant interactions for SSBs and SES over-time. However, we acknowledge the reviewers comment so we have added in more information

“Upon further inspection, this steady increase reflects the increase in reports of ‘Never’ consumption over the years (see Supplementary table 15).” (Lines 235-236).

18. Are you able to provide actual p-values as opposed to reporting as p<0.05; appreciate you have CI but if using the p-value too then would be better.

**Response**: Where possible actual p-values have been added throughout the paper or have been altered to show ‘<0.001’.

19. Re presentation of results: use more detailed subheadings, for example, as the effect of year on both SSBs and ED; and then another subheading noting the effect of gender, SES and year group and just highlight the key points.

**Response**: We thank the reviewer for this comment. We acknowledge that the use of subheadings in the results section helps guide the reader and we have made these changes in the results section.

Discussion

20. Can the authors provide some detail as to why they think there has been such a reduction in daily SSBs since 2002/9 – here there was a steep reduction in comparison to other survey periods, prior to the sugar tax etc. Anything related to changes in school food and drink purchasing (relates back to a prior question about is this data total dietary data?) Switch from SSB to diet drinks?

**Response:** To reflect the reviewer’s suggested changes, we have added the following information to the manuscript “A number of environmental and policy changes may have contributed to the observed reduction in daily SSB consumption between 2000 and 2009. In 1996, the UK was reported to have one of the highest proportions of overall food advertisements worldwide, with 79% of adverts devoted to sweet or high fat foods. Since, more stringent advertising guidelines have been introduced to reduce young people’s exposure to advertising of unhealthy food products. Between 2001 and 2007, a series of school food policies were also introduced across Wales in an attempt to improve the nutritional standards of food and drink provided in schools [36, 37]” (Lines 363-370)

Limitations:

21. Need to acknowledge point about the SES coding, change from 2002,

**Response**: As per our response above, this has since been acknowledged.

22. I wonder if the authors can comment on the robustness of the SES categorisation, in particular for this piece of research? I.e. possible changes in the ‘sensitivity’ of FAS over the time periods. In addition, the paper referenced (Currie et al, 2008) states “FAS associations are strong for health out-comes that are related to family culture and behaviour, but less so for some behaviours where peer norms area potentially powerful influence”. I wonder if the authors can comment on this in terms of the influence of peers in SSB consumption and the subsequent use of FAS to categorise SES?

**Response**: We thank the reviewer for this insightful question. While peer influence has a strong influence on behaviours, the FAS is widely used in research to capture socioeconomic differences. However, we accept that peer influence may become stronger over-time, particularly when the young person becomes older and more autonomous. However, health behaviours are often adopted in childhood and are, to some extent, driven by family SES which provides a justification of using FAS in the models.

23. Acknowledge the SSB drinks aren’t categorised into different sugar contents, cross sectional survey – not individual-child changes. Acknowledgment of the limitations of FFQs (in adolescents) should be included

**Response**: Thank you for raising these limitations. We have an existing line in the limitations section which acknowledges the cross-sectional design of the survey (line 421). We have however, edited an existing sentence to reflect the sugar content across SSBs and EDs; “Lastly, while SSBs and EDs are increasingly varied in terms of amounts of sugar and ingredients included, our single item measures treat these as homogeneous products” followed by an additional sentence “Our ability to comment on the content or volume of drinks consumed is limited, with a reliance on consumption frequency data only.” (Line 428-430)

24. Line 313: What does “other HBSC countries” mean? Is the same survey conducted in other countries? Just needs a brief explanation, or perhaps include more detail about the HBSC and SHRN surveys in the methods (as mentioned above).

**Response**: Thank you for your comment. We have added the following information to our methods section “The HBSC survey, a collaborative cross-national survey, is administered every four years and currently involves 50 countries and regions across Europe and North America. The SHRN survey is administered every two years and is based on the HBSC survey allowing integration of the two surveys every four years.” (Lines 118-122)

25. Two other questions: does the model account for the variation in sample size? For example, in one of the later surveys roughly over 40,000 by comparison to much smaller survey numbers previously?

**Response**: We thank the reviewer for this comment. We have not accounted for the variation in sample size in the model, and as the reviewer rightly points out that the sample sizes do vary considerably over-time. From this, we have acknowledged this in the limitations section; “Fourth, we do not account for the variation in sample size which increases over-time.” (Line 426).

**Reviewer 2 comments**

1. My main concern is around the grouping of response options. Around 50% of responses were categorised as ‘weekly’, defined as consuming SSB/ED between 1 and 6 times per week. There is a big difference between these frequencies. This should be discussed as a limitation, and the disaggregated response data provided as a supplementary table to assist with interpretation of findings. I have given some more specifics to this (points 5 and 7 below), along with some other minor recommendations (in order of appearance through the manuscript).

**Response**: Thank you for your supportive comments and for highlighting key concerns around data categorisation. As outlined within our responses below, we have provided further elaboration within our limitations section and responded to individual comments below.

2. The aim should be clarified, to differentiate between the amount of SSB and ED data you have (i.e. 19 years vs 5 years), and the addition of the word ‘frequency’, to describe the consumption pattern that you are measuring.

**Response**: Thank you for your suggestion. In line with reviewer 1’s comment also, we have updated the text to read “The aim of this study is to examine the consumption frequency of SSBs and EDs among 11-16 year olds over time. With use of national data collected between 1998 and 2017, we examined overall consumption and reports among sociodemographic subgroups. Data available for SSB consumption spanned a 20-year period (1998-2017) and ED consumption across five years (2013-2017).” (Lines 104-109)

3. In the methods, please give a brief overview of the study sampling strategies and procedures for each survey; including sampling methods, frequency of administration and year groups sampled at each time point. Were the same schools sampled every time, or new schools approached at each data collection wave? Also describe the approach that led to larger sample sizes at the 2015 and 2017 waves.
   **Response**: We thank the reviewer for this observation. As the survey is used considerably in Wales, we believe that the references to other studies with more detailed information is acceptable as readers can access this information if they so wish. We have added further information regarding the frequency of administration along with wider details requested by Reviewer 1. (Lines 116-123). We have also highlighted the changes in sample sizes as requested, as this is an important point noted within our limitations section: “Fourth, we do not account for the variation in sample size which increases over-time.” (Line 426)
4. Please also indicate in the methods whether questionnaires were completed solely by the students, a parent proxy or a combination.

**Response**: To provide greater clarity, the following text has been added “Student self-report data from…” (Line 114)

5. In the results (line 211) please revise the statement ‘remained stable at 6% each year’ – ED data were collected at 3 time points in the period, not each year.
   **Response**: Thank you, to reflect this, we have removed ‘each year’ from the current sentence.
6. Could the disaggregated SSB & ED response data be made available as a supplementary table and mentioned in your results? In particular, it would be useful to see the spread of intake responses that were grouped into the ‘weekly’ category at each time point.
   **Response**: We thank the reviewer for this comment. We have now provided this information in a series of supplementary tables (S13-S16). As the reviewer can see for SSB’s, the increase in weekly SSB use is mostly from the ‘Once a week’, and ‘2 – 4 times a week’ categories. For SSB daily use both categories of ‘Every day, once a day’ and ‘Every day, more than once a day’ have both decreased over-time. For ED use, all weekly use has decreased across the ‘Once a week’, ‘2 – 4 times a week’ and ‘5 – 6 times a week’ categories. Likewise, ED use has remained stable over 2013 – 2017 for the ‘Every day, once a day’ and ‘Every day, more than once a day’ categories. However, ‘Less than once a week’ ED use has increased, it is ‘Never’ use of ED that accounts for the increase in ‘Never or less than weekly use’. In addition to the supplementary tables, we have added this information in the paper, please see under the subheading ‘Time Trends’. (Lines 221-222 and 235-236)
7. The abstract and discussion both mention that the data is ‘nationally representative’, but this is not established in the methods or results. Please do so in at least one of those sections, and then add a comment to the discussion about the representativeness of the samples and generalisablty of your findings.
   **Response**: For the more recent results, we show that data from nearly all secondary schools in Wales are captured. However, as the reviewer rightly states, this is not substantiated in the previous years (<2013) so this statement has been removed.
8. Please add a discussion point about whether these categorical groupings might lead to an overestimation of the scale of the decrease in SSB consumption. Moving from daily to weekly SSB consumption is a great outcome, however the true decline may be much more modest. For example, is it possible that this decline is the result of SSB intakes decreasing from 7 to 6 times per week? Is it also possible that this is reflected by the replacement of one of the SSBs per week with an ED? The recommended disaggregated data table (see point 5) might help you explore the likelihood of this limitation.

**Response**: We thank the reviewer for this important point. We have discussed this in the Time Trends section, and we also have acknowledged this as a limitation; “Fifth, as only three categories are used, some detail is lost, but trends have been explored in Supplementary Tables S13 – 16.” (Lines 427-428)

9. A further limitation to consider is the reporting of only frequency rather than incorporating the amount consumed at any occasion. As portion sizes of commercial foods appear to have increased over time, is it possible that total SSB consumption (eg in mls) has not declined?

**Response**: Thank you for your comment. In line with the above suggestions, we have added further information to our limitations section drawing on the earlier work of Church (2008) and Benton (2015) who highlight the difficulty in making generalizations about portion size over time, with unclear UK trends. This has been reflected in the following text; “Our ability to comment on the content or volume of drinks consumed is limited, with a reliance on consumption frequency data only. This poses potential implications for the interpretation of our findings as despite a common perception that portion sizes have widely increased [43], UK trends in soft drink portion sizes over time remain unclear [44]. (Lines 430-434).
